# Clock Genes in Glia Cells: A Rhythmic History

**DOI:** 10.1177/1759091416670766

**Published:** 2016-09-25

**Authors:** Donají Chi-Castañeda, Arturo Ortega

**Affiliations:** 1 Centro de Investigación y de Estudios Avanzados del Instituto Politécnico Nacional, Ciudad de México, México; 2 Soluciones para un México Verde, S.A de C.V., Santa Fé Ciudad de México, México

**Keywords:** circadian rhythms, clock genes, glia cells, oscillators, rhythmicity, suprachiasmatic nucleus

## Abstract

Circadian rhythms are periodic patterns in biological processes that allow the organisms to anticipate changes in the environment. These rhythms are driven by the suprachiasmatic nucleus (SCN), the master circadian clock in vertebrates. At a molecular level, circadian rhythms are regulated by the so-called *clock genes*, which oscillate in a periodic manner. The protein products of clock genes are transcription factors that control their own and other genes’ transcription, collectively known as “clock-controlled genes.” Several brain regions other than the SCN express circadian rhythms of clock genes, including the amygdala, the olfactory bulb, the retina, and the cerebellum. Glia cells in these structures are expected to participate in rhythmicity. However, only certain types of glia cells may be called “glial clocks,” since they express PER-based circadian oscillators, which depend of the SCN for their synchronization. This contribution summarizes the current information about clock genes in glia cells, their plausible role as oscillators and their medical implications.

## Introduction

Most light-sensitive organisms have built-on time-measuring devices that are commonly known as circadian clocks. These structures allow them to anticipate day time and hence to organize their behavior as well as physiological and biochemical processes in a proactive manner. Circadian rhythms are generated endogenously through genetic control (King and Takahashi, 2000) in living systems, ranging from bacteria to humans (Harmer et al., 2001; Bell-Pedersen et al., 2005); and control vital aspects of the organism physiology, from sleeping and waking to neurotransmitter secretion and cellular metabolism. At the center of these rhythms resides the circadian clock machinery, an amazingly transcription-translation feedback system regulated by a group of genes that oscillate in a circadian manner, the so-called *clock genes*. The circadian system is hierarchically organized, meaning that while molecular oscillations occur in most cells and tissues of the body, the suprachiasmatic nucleus (SCN) functions as the master regulator to synchronize the phase of the other oscillating tissues (Schibler and Sassone-Corsi, 2002; Hastings et al., 2008). Although the general consensus of the cellular identity of oscillating cells in the brain point to neurons, glia cells of different brain areas have been proposed to act as circadian oscillators that are dependent on the SCN for their synchronization (Siwicki et al., 1988; Zerr et al., 1990; Ewer et al., 1992). Nevertheless, despite of the fact that glia cells have a pivotal role in most of the central nervous system (CNS) functions, their role in circadian physiology is only begging to be understood. With this in mind, we discuss here the recent knowledge about clock genes in glia cells, their plausible role as cellular oscillators, and their involvement in pathological conditions.

## Circadian Rhythms

The term *circadian* was introduced by Halberg to describe the biological rhythms that have a period of approximately 24 h, namely the circadian rhythms (from the Latin *circa*, “around,” and *dies*, “day,” meaning literally “about a day”; Halberg, 1959). Circadian rhythms are found in every kingdom of life, and in mammals, regulate a plethora of functions in the organism, including the rest-activity cycle, daily variations in metabolism and body temperature, and the rhythmic secretion of hormones (Stratmann and Schibler, 2006).

In higher vertebrates, circadian oscillators exist in the brain as well as in other organs or tissues (Tosini and Menaker, 1996; Granados-Fuentes et al., 2006). The “master clock” that coordinates the activities of other oscillators resides in the SCN, which is located in the anterior hypothalamus and is comprised of a heterogeneous population of neurons and relatively understudied glia. Circadian oscillators in other brain areas or tissues are called “peripheral clocks” and are under the influence of the SCN, presumably through combination of neural and humoral signaling (Balsalobre et al., 2000; Cheng et al., 2002; Schibler and Sassone-Corsi, 2002; Chung et al., 2011).

The SCN receives photic information from the environment via neurons transcending from the retina through the retino-hypothalamic tract (Moore and Lenn, 1972), which allows the setting of SCN circadian oscillators to external light cues (Johnson et al., 1988). Particularly, the surgical ablation of the SCN in mammals causes animals to become arrhythmic in locomotor activities, endocrine output, and other biochemical and physiological processes (Moore and Eichler, 1972; Stephan and Zucker, 1972; Turek, 1985). Transplantation of SCN tissue to SCN-lesioned animals restores circadian rhythms with the period of the donor (Ralph et al., 1990; Sujino et al., 2003). When isolated *in vitro*, the SCN continues to express circadian rhythms in glucose metabolism, gene expression, and electrical activity similar to the *in vivo* scenario (Green and Gillete, 1982; Herzog et al., 1997; Yamazaki et al., 2000).

## Molecular Machinery of Circadian Clocks

The molecular mechanism that generates circadian rhythms involves the interaction positive and negative feedback loops of transcriptional or translational processes of clock genes (Dunlap, 1999; Harmer et al., 2001; Reppert and Weaver, 2001). In mammals, two basic helix-loop-helix transcription factors, Circadian Locomotor Output Cycles Kaput (CLOCK) and Brain and Muscle Aryl Hydrocarbon Receptor Nuclear Translocator-Like Protein 1 (BMAL1), heterodimerize and subsequently bind to conserved E-box sequences in target gene promoters. In this manner, this complex controls the rhythmic expression of mammalian *Period* (*Per1, Per2, Per3*) and *Cryptochrome* (*Cry1, Cry2*) genes (Dunlap, 1999; Reppert and Weaver, 2001). If the concentration of these proteins is large enough, they dimerize and inhibit transcription of the genes *Per1* y *Per2* interacting with CLOCK and BMAL1. The positive feedback loop is mediated PER2, regulating *Bmal1* transcription; BMAL1 promotes heterodimerization of CLOCK:BMAL1, so that transcription cycles *Per*/*Cry* can be restarted (Dunlap, 1999; Harmer et al., 2001; Reppert and Weaver, 2001; Okamura et al., 2002).

**Figure 1.**
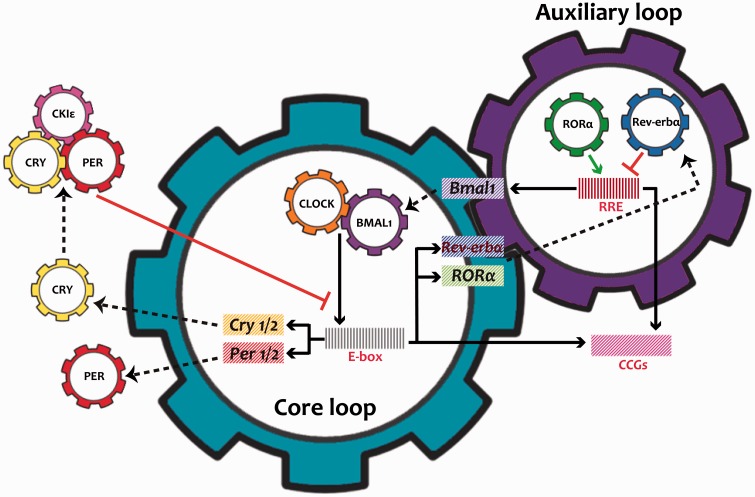
Molecular mechanisms of the clock. The mammalian circadian oscillator is composed of an autoregulatory transcriptional network with two interlocked feedback loops: core and auxiliary. The CLOCK/BMAL1 heterodimer, the integral component of the core loop, induces E-box mediated transcription of the negative regulators *Periods* (PERs) and *Cryptochromes* (CRYs). Accumulated PER and CRY proteins intensively repress E-box mediated transcription until their levels have sufficiently decreased. Additionally, another regulatory loop is induced by CLOCK:BMAL1 activating transcription of the nuclear receptors *RORa* and *Rev-erba*, which modulate *Bmal1* mRNA levels by competitive actions on the RRE element residing in the *Bmal1* promoter. Collectively, the cycling of the clock components also determines the levels of the *clock-controlled genes* (CCGs) by transcription via the E-box or RRE to achieve their oscillating patterns and thus to generate rhythmic physiological output.

Another regulatory loop is mediated by the orphan nuclear receptors, the Retinoic Acid Receptor-Related Orphan Receptor *α/β/γ* (*ROR α/β/γ*) and the Reverse Erb *α/β* (*Rev-erb α/β*), that are responsible to activate and inhibit, respectively, transcription of *Bmal1* through the retinoic acid Receptor Response Element (RRE) in its promoter, leading it to oscillate in a circadian manner (Figure 1; Preitner et al., 2002; Sato et al., 2004; Akashi and Takumi, 2005; Guillaumond et al., 2005).

In addition to the core regulation at the level of transcription or translation, circadian clock proteins are also subjected to extensive posttranslational modifications that appear to control their cellular localization, protein stability, and activity. For example, Casein Kinase Iɛ and δ (CKIɛ/δ) are known to be critical factors that regulate the turnover of PERs and CRYs in mammals (Akashi et al., 2002; Eide et al., 2002; Gallego and Virshup, 2007); however, kinase CKIɛ also activates BMAL1-mediated transcription (Eide et al., 2002).

Importantly, circadian transcription factors not only regulate their own transcription but also regulate the expression of numerous other *clock-controlled genes* (CCGs; Dunlap, 1999; Reppert and Weaver, 2001). In fact, it is currently estimated that approximately 43% of the mammalian genome is rhythmic, and these CCGs are involved in a wide array of physiological functions throughout the body and the brain (Zhang et al., 2014). It is noteworthy that CCGs are rhythmically regulated by the circadian clock, but differ from clock genes, in that their protein products are not essential for function of the clock. Among the genes that are under circadian regulation included metabolic enzymes, like phosphoenolpyruvate carboxykinase (Phillips and Berry, 1970); ion channels, like cGMP-gated cation channels, various voltage-gated calcium and potassium channels, the Na^+^/K^+^-ATPase, and a long-opening cation channel (Ko et al., 2009); and peptides, like Arginine-Vasopressin (AVP; Jin et al., 1999) and DBP (D element-Binding Protein; Le Martelot et al., 2009).

## Glia Cells

In all parts of the nervous system, glia cells outnumber neurons, and they make up a large part of nervous tissue. For instance, it is known that glia cells occupy about half the volume of the brain. These cells have critical roles in modulating synaptic transmission, plasticity, and behavior, in addition to their well-characterized functions in synapse development and neurodegeneration (Jessen and Richardson, 2001; Jessen, 2004; Stork et al., 2012; Clarke and Barres, 2013; Brown and Neher, 2014). However, astrocytes also regulate physiologically neuronal circuits in the adult brain that control neuronal excitability, cognitive state (Lee et al., 2014), and responses to drugs of addition (McIver et al., 2012; Turner et al., 2013).

The term *glia* is derived from the Greek word *glia*, which literally means “glue.” In 1858, Rudolf Virchow described to the glia cells as a connective tissue that binds nervous elements together (Virchow, 1858). Soon after, in 1870s, the cellular nature of glia was firmly established by Camillo Golgi (1873). The term *glia cells* denotes in fact a broad category of cells that is made up of many subtypes; accordingly, there are three types of glia cells in the mature CNS: astrocytes, which are important for the extracellular ion homeostasis, neurotransmitter recycling of the major excitatory amino acid (Danbolt et al., 2016), and regulation of complex brain mechanisms, such as sleep homeostasis (Halassa et al., 2009) and memory (Newman et al., 2011; Suzuki et al., 2011; Han et al., 2012; Stehberg et al., 2012); oligodendrocytes, key factors in neuronal conductivity, and in which their own biology, myelination, and maintenance of myelin sheaths are very complex processes (Baumann and Pham-Dinh, 2001; Barres, 2008; Watkins et al., 2008) that their disturbances are associated with major diseases of the nervous system; and finally, microglia cells, the *brain-immune cells* that also have relevant roles in the maintenance of neuronal circuitry, regulation of behavior (Hayashi et al., 2013b), and functional state of neurotransmission (Hayashi et al., 2013a). Remarkably, it has also been described that all of these three types of glia cells also play an important role in the regulation of circadian rhythms (Prosser et al., 1994; Li et al., 2002; Matsumoto et al., 2011; Ng et al., 2011; Fonken et al., 2015).

## Clock Genes in Glia Cells

### Astrocytes

The biochemical characterization of clock genes has allowed the identification of brain areas that possess the molecular machinery needed for the generation of circadian rhythms. Consequently, daily oscillations in gene expression of clock genes have been identified in a number of brain regions (Feillet et al., 2008), including the cerebellum (Akiyama et al., 1999; Namihira et al., 1999), amygdala, olfactory bulb, the lateral *habenula*, and a variety of nuclei in the hypothalamus (Guilding and Piggins, 2007). Interestingly in these areas, clock gene expression is by no means restricted to neurons but is not uncommon to detect them in the most abundant cell type in the CNS: glia cells, which show circadian rhythms *in vivo* and *in vitro* (Siwicki et al., 1988; Zerr et al., 1990; Ewer et al., 1992; Yagita et al., 2010; Ng et al., 2011; Fonken et al., 2015).

The first work to suggest that glia may contain molecular oscillators was reported in 1990; it was demonstrated that the canonical clock protein PER was localized both in neurons and glia cells of the fly brain, and that it showed robust circadian rhythms and abundance in both cell types (Zerr et al., 1990). Soon after, using genetic mosaic analysis, it was reported that certain weakly rhythmic flies contained detectable PER only in glia; this was interpreted as an evidence for a role of glial oscillators in the pacemaker driving rhythmic behavior (Ewer et al., 1992). Thereafter, other studies in rat and mouse astroglia demonstrated rhythmic expression of clock genes in astrocytes, indicating that these cells contain a PER-based molecular oscillator that damps in the absence of neuronal signals (Prolo et al., 2005; Yagita et al., 2010). Interestingly, astroglial cultures were capable to display a sustained rhythmicity (7 days or longer) when cocultured with SCN explants, whereas cortical explants did not influence rhythmicity (Prolo et al., 2005); suggesting that a secreted neuronal factor expressed in the SCN may be required for sustained rhythms in glia cells.

Several studies have explored the role of the mammalian PER-based oscillator in regulating glial physiology. Hence, it has been reported that there is a diurnal rhythm in *Glast* (glutamate/aspartate transporter) glutamate (Glu) transporter gene expression and protein amount within the SCN with the peak protein occurring at the beginning of the photoperiod in an light:dark (12:12) cycle, in spite of the fact that it was not determined whether this rhythm persists or not in conditions of constant darkness or constant light (Spanagel et al., 2005). Moreover, the observation that GLAST levels do not show an obvious rhythmicity in *Per2* mutant mice suggests the presence of a circadian control (Spanagel et al., 2005). Years later, it was demonstrated that cultured cortical astrocytes from clock mutant animals have reduced *Glast* mRNA and protein levels (Beaulé et al., 2009). These results suggest that the vast majority of Glu uptake activity (glial) is a function of the transcription factors *Clock* and *NPAS2* and of the transcriptional regulator *Per2* (Beaulé et al., 2009). Such dependence could be explicated by the involvement of CLOCK and NPAS2 in *Glast* transcription indirectly or in GLAST protein stability or localization (Danbolt, 2001). Beaulé’s study in 2009 also showed that despite of the presence of circadian rhythms in *Per* gene expression in cultured astroglia, no evidence was found for circadian changes in Glu uptake; so that a noncircadian role for clock proteins might be involved in the regulation of *Glast* gene transcription or *Glast* mRNA translation and stability (Beaulé et al., 2009). Accordingly, Morioka et al. (2012) reported no circadian-mediated GLAST expression in mice spinal cord; however, both of these conflicting results could be explained in terms of a differential tissue regulatory mechanisms of circadian-controlled molecules expression (brain vs. spinal cord) and even a loose of molecular components of the glia clock in cultured astrocytes.

Regarding Glu, it is known that this neurotransmitter participates in photic entrainment of circadian rhythms, so it is important to mention that Glu regulates the clock protein BMAL1 in primary cultures of chick cerebellum Bergmann glia cells. In that study, a Glu-driven dose and time-dependent BMAL1 increased expression was reported, being this phenomena the result of a stabilization of the protein after it has been phosphorylated by PKA or PKC kinases; pointing out that Glu is critically involved in glia BMAL1 expression that these cells are important in the control of circadian rhythms in the cerebellum (Chi-Castañeda et al., 2015).

Another important finding is the discovery of high-amplitude daily rhythms in the distribution of glial fibrillary acidic protein (GFAP, a specific astrocyte marker in the adult brain) in astrocytes of the SCN (Lavialle and Serviere, 1993). These rhythmic patterns persist in constant darkness in the SCN of hamsters, rats, and mice (Lavialle and Serviere, 1993; Moriya et al., 2000) suggesting that these rhythms are intrinsic and independent of external light cues. Although, the role of daily oscillations in GFAP immunoreactivity is unknown, it has been seen noticed that mice lacking the *Gfap* gene show impaired long-term depression in the cerebellum, as well as reduced eyeblink conditioning (Shibuki et al., 1996), indicating that GFAP in glia cells has some role in regulating neuronal function. Subsequently, Leone et al. (2006) demonstrated a daily variation of GFAP in the mouse SCN; however, the authors suggested that these oscillations reflect a response of astrocytes in the SCN to inputs from the immune system via signaling through the immune-related transcription factor nuclear factor-κB (NF-κB).

Astrocytes communicate with nearby neurons by a process known as gliotransmission (Haydon, 2001; Fields and Burnstock, 2006; Perea et al., 2009), being adenosine triphosphate (ATP) and Glu, the best known transmitters released by these cells (Parpura and Zorec, 2010). *In vivo*, circadian rhythms in ATP release appear to derive primarily from astrocytes within the SCN (Womac et al., 2009). The mechanisms responsible for generating ATP oscillations in SCN cells and cortical astrocytes are unknown; however, calcium-dependent signaling is likely to be involved in extracellular ATP accumulation and its circadian profile (Womac et al., 2009). The functional implications of extracellular ATP rhythms have not been described yet, but probably this nucleotide participates in intracellular signaling between circadian oscillators in the SCN and other brain regions. More recently, it was demonstrated that astrocytes display daily extracellular ATP oscillations that depend on key clock genes (*Clock*, *Per*, and *Bmal1*) and inositol triphosphate (IP_3_) signaling (Marpegan et al., 2011). Thus, these results indicate that extracellular ATP levels are augmented at specific times of day and suggest a clock-induced increase in energy metabolism and glia activity, which may participate in sleep-wake changes in the brain (Marpegan et al., 2011). Remarkably, astrocytes in the SCN respond to photic stimulation with an increase in FOS expression (Bennett and Schwartz, 1994), suggesting their involvement in the response to light and, possibly, entrainment. Moreover, cultured astrocytes respond to nanomolar concentrations of vasoactive intestinal polypeptide (VIP) with clock gene induction, ATP release, and shifts in their circadian rhythms (Marpegan et al., 2009; Marpegan et al., 2011).

Subsequently, it was proved that glia cells of the adult brain could physiologically modulate circadian neuronal circuitry and behavior through glia calcium signaling (Ng et al., 2011). Genetic manipulations of glia vesicle trafficking, the membrane ionic gradient, or internal calcium stores all lead to arrhythmic locomotor activity in Drosophila, in which a single type of glia cells, the astrocytes, are relevant for the circadian modulation of behavior; thereby, glia Ca^2+^ signaling is critical for the modulation of the neuronal circadian circuitry (Ng et al., 2011). It should be noted that the Drosophila astrocytes and mammalian brain are remarkably similar in regard to their morphology and molecular signatures, further suggesting a conservation of function.

It has been also demonstrated that spinal cord circadian expression of clock genes is dependent of the activity of astrocytes, suggesting the involvement of circadian rhythmicity in various spinal functions, including nociception (Morioka et al., 2012). Therefore, the intensity or presence of pathological pain and the efficacy of a certain pain treatment could vary significantly depending on the time of day. Moreover, a circadian oscillation *Glutamine synthetase* (GS) mRNA in the spinal cord was also documented; being this relevant for the Glu-Glutamine metabolic cycle, and the amount of Glu repackaged in the primary efferent neuron terminals located in the dorsal horn of spinal cord may change within a day (Morioka et al., 2012).

### Microglia

The discovery about a molecular clock in the microglia is relatively recent. Hayashi et al. (2013b) reported the first evidence that cortical microglia contain an intrinsic molecular clock, which regulates diurnal changes of its morphological aspect. Microglia, in contrast to astrocytes, regulates the sleep–wake cycle-dependent changes in synaptic strength through the extension and retraction of their processes (Hayashi et al., 2013a). Cortical microglia exhibits a circadian expression of Cathepsin S (CatS), a microglia-specific lysosomal cysteine protease in the brain, which is regulated by CLOCK-BMAL1-driven transcriptional negative feedback loops. Interestingly, when CatS suffers a disruption induces hyperlocomotor activity due to failure to downscale the synaptic strength during sleep, which is necessary for the acquisition of subsequent novel information after waking (Hayashi et al., 2013b); therefore, it is possible that dysfunction of the microglial intrinsic circadian clock could play a causative role in neuropsychiatric disorders based on sleep disturbance, including depression and cognitive impairment (Bhattacharjee, 2007; Hayashi et al., 2014).

Recently, it was reported that microglia possess circadian clock mechanisms and display rhythmic fluctuations in basal inflammatory gene expression (including IL1β, TNFα, IL6, and IL1R1) as well as inflammatory potential, in which time-of-day differences in microglia priming appear functionally relevant as they are reflected in circadian differences in sickness response (Fonken et al., 2015). Of relevance is to note that rhythms in microglia oscillate in the absence of glucocorticoids, so that one limitation is the isolating microglia only 24 hr after corticosterone removal does not eliminate possible priming effects of glucocorticoids (Frank et al., 2014); since glucocorticoids can phase shift peripheral circadian clock (Sujino et al., 2012).

### Oligodendrocytes

This type of cell is the least studied of the three kinds of glia cells. To date, there is no report showing that oligodendrocytes have an internal circadian clock; however, the information indicates that clock genes might regulate oligodendrocytes precursor cells (OPCs) proliferation in the hippocampus (Matsumoto et al., 2011). The OPCs give rise to mature oligodendrocytes, which contribute to axonal myelination and to mature neurons in the piriform cortex in the adult rodent brain (Dimou et al., 2008; Rivers et al., 2008); therefore, oscillatory proliferation of OPCs might have great impact on hippocampal function because OPC proliferation itself, in response to neuronal activity, may eventually modulate the synaptic plasticity for the hippocampus.

## Clinical Implications

It has been documented that glia cells are involved in most types of brain pathologies from acute lesions to chronic neurodegenerative processes and psychiatric diseases. With this in mind and regarding to clock genes, it is known that absolute expression levels of these genes are modulated under pathological conditions (Aston et al., 2004; Benedetti et al., 2008; Beaulé et al., 2009; Soria et al., 2010; Gu et al., 2015). Specifically, a nonfunctional *Per2* gene leads to GLAST reduced expression and as consequence Glu uptake is diminished and a hyperglutamatergic state is triggered (Spanagel et al., 2005; Yuferov et al., 2005). Astrocytic Glu release has clear pathophysiological implications, ranging from ischemic lesion such as stroke, to white matter injury through demyelinating disorders like multiple sclerosis, and to dementias such as Alzheimer’s and Huntington diseases (Domingues et al., 2010). Moreover, laboratory studies have shown that Glu also modulates the levels of dopamine and other neurotransmitters and neuropeptides that mediate both positive and negative aspects of drug reinforcement and reward. Both hyper- and hypoglutamatergic states in specific brain regions are associated with different stages of addiction, including development, persistence, and abstinence. Interestingly, clock genes seem to be involved in the modulation of common mechanisms of drug abuse-related behaviors (Yuferov et al., 2003). Notably, haplotypes of the *Per2* gene have been associated to the amount of alcohol consumption in alcoholic patients, suggesting that altered function in this gene leads to changes in alcohol reinforcement processes (Spanagel et al., 2005).

In relation with *Per1*, *Per3*, and *Bmal1*, alterations in these genes result in reactive oxygen species imbalance and chronic oxidative stress in the brain, modifications in both short- and long-term memory, as well as association with diverse psychiatric diseases like schizophrenia, Parkinson, bipolar disorder, and Alzheimer (Aston et al., 2004; Nievergelt et al., 2006; Benedetti et al., 2008; Mansour et al., 2009; Krishnan et al., 2009; Gerstner, 2010; Gu et al., 2015; Song et al., 2015). However, a single clock gene is not related with a particular medical condition, but it has been reported that a single clock gene can have different repercussions on health, and several clock genes may be related to the same pathology. Such is the case of the clock genes *Npas2*, *Gsk3β, Dbp*, *Cry1*, and *Clock* that are also implicated in psychiatric diseases (Niculescu et al., 2000; Bhat and Budd, 2002; Johansson et al., 2003; Takano et al., 2004; Roybal et al., 2007; Soria et al., 2010; Geoffroy et al., 2015) as well as in other brain pathologies (Table 1).

**Table 1.** Clock Genes and Their Implications in Brain Pathologies.

Clock gene	Preparation	Pathophysiological implications	References
*Per1*	*Per-null mutant (flies) Per-null mutant (flies) Per1* mutant animals (mice) Post-mortem tissue from Brodmann’s area 21 (humans) DNA samples (humans)	Regulates reactive oxygen species homeostasis and chronic oxidative stress in the brain Long-term memory formation Modulates cocaine sensitization Schizophrenia Association with Parkinson’s disease	Krishnan et al. (2009) Sakai et al. (2004) Abarca et al. (2002) Aston et al. (2004) Gu et al. (2015)
*Per2*	*Per2* mutant animals (mice) Astrocytes in culture (mice) Whole blood (humans)	Modulates cocaine sensitization Influences the glutamatergic system (decreases GLAST) and modulates alcohol consumption Modulates alcohol consumption	Abarca et al. (2002) Spanagel et al. (2005) Spanagel et al. (2005)
*Per3*	DNA samples (humans)	Association with bipolar disorder	Nievergelt et al. (2006) Benedetti et al. (2008)
*Clock*	Nerve cord and brains (*Drosophila*) *Clock* mutant (mice) *Clock* mutant (mice) Astrocytes in culture (rat and mouse) or Single nucleotide polymorphisms (humans) Single nucleotide polymorphisms (humans)	Regulates cocaine sensitization (regulator of tyrosine decarboxylase) Behavioral profile that is strikingly similar to human mania Regulates dopaminergic activity (regulating behavior and mood) Association with bipolar disorder Influences the glutamatergic system (decreases GLAST)	Andretic et al. (1999) Roybal et al. (2007) Roybal et al. (2007) Benedetti et al. (2003), Soria et al. (2010) Beaulé et al. (2009)
*Cry 1*	Single nucleotide polymorphisms (humans)	Association with unipolar major depressive disorder	Soria et al. (2010)
*Dbt*	Nerve cord and brains (*Drosophila*)	Regulates cocaine sensitization (regulator of tyrosine decarboxylase)	Andretic et al. (1999)
*Rev-erbα*	Cortical microglia and myeloid cell lineage (mice), and buffy coats (humans) Cerebellar tissue (mice)	Modulates innate immune responses (inflammatory diseases, for example IL-6 in rheumatoid arthritis) Development delays in cerebellum, delayed migration of granule cells, and increased apoptosis of neurons	Gibbs et al. (2012) Nakanishi (2014) Chomez et al. (2000)
*Bmal1 Cyc*	DNA samples (humans) *Bmal1* knock-out (mice) Brain tissue extracts of *Bmal1* knock-out (mice) DNA samples (humans) Hippocampal cells (mouse) Nerve cord and brains (*Drosophila*)	Association with bipolar disorder Regulates both short- and long-term memory Regulates reactive oxygen species homeostasis and chronic oxidative stress in the brain Association with Parkinson’s disease Association with Alzheimer’s disease Regulates cocaine sensitization (regulator of tyrosine decarboxylase)	Mansour et al. (2006) Nievergelt et al. (2006) Mansour et al. (2009) Gerstner (2010) Kondratova et al. (2010) Gerstner (2010) Kondratova et al. (2010) Gu et al. (2015) Song et al. (2015) Andretic et al. (1999)
*Npas2*	Genomic DNA samples (humans) Astrocytes in culture (rat and mouse) Genomic DNA samples (humans) Single nucleotide polymorphisms (humans)	Seasonal affective disorder Influences the glutamatergic system (decreases GLAST) Association with bipolar disorder Association with unipolar major depressive disorder	Johansson et al. (2003) Beaulé et al. (2009) Geoffroy et al. (2015) Soria et al. (2010)
*Csnk1ɛ*	Genomic DNA samples (humans)	Circadian rhythm sleep disorders	Takano et al. (2004)
*Gsk3β*	Genomic DNA samples (humans)	Implicates in several CNS disorders, such as affective disorders, schizophrenia, and Alzheimer’s disease Age of onset and presence of psychotic symptoms in bipolar disorder	Bhat and Budd (2002) Benedetti et al. (2004) Lee and Kim (2011)
*Dbp*	Specific brain regions (humans)	Mania and psychosis	Niculescu et al. (2000)

*Note. Per 1–3*, period 1–3; *Clock*, circadian locomotor output cycles kaput; *Cry 1*, cryptochrome 1; *Dbt*, double time; *Rev-erbα*, reverse Erb alpha; *Bmal1*, brain and muscle ARNT-like protein 1; *Cyc*, cycle; *Npas2*, neuronal PAS domain protein 2; *Csnk1ɛ*, casein kinase 1 epsilon; *Gsk3β*, glycogen synthase kinase 3 beta; *Dbp*, D site of albumin promoter binding protein.

Particularly, the disturbances in sleep parameters have received limited attention in spite of the fact that they are associated with a spectrum of neurological and psychiatric disorders. We know that sleep patterns are affected not only by independent homeostatic mechanisms that determine the amount of sleep required (Borbely and Achermann, 1999) but also by circadian timing mechanisms. Accordingly, mutations in clock genes, including *Clock*, *Bmal1*, and *Cry1/2*, result in alterations in sleep time, sleep fragmentation, and atypical responses following sleep deprivation (Naylor et al., 2000; Wisor et al., 2002; Laposky et al., 2005).

However, these sleep disruptions also have profound effects in the immune system altering the number of circulating lymphocytes, natural killer cells, antibody titers, and levels of cytokines (Meier-Ewert et al., 2004; Vgontzas et al., 2004; Everson, 2005; Hui et al., 2007) that translate into impaired immune function when an immune challenge is presented (Irwin et al., 1996; Born et al., 1997). The importance of the immune cells lies in that they exhibit circadian expression of clock genes which in turn are involved in regulating immunological activities. For example, *Rev-erb* gene represses macrophage gene expression (Lam et al., 2013) and targets inflammatory function of macrophages through the direct regulation of Ccl2 (Sato et al., 2014). Additionally, *Bmal1* controls rhythmic trafficking of inflammatory monocytes to sites of inflammation (Nguyen et al., 2013). Hence, circadian disruptions exacerbate inflammatory responses in both periphery (Castanon-Cervantes et al., 2010) and CNS (Fonken and Nelson, 2013).

It is noteworthy that, in relation with microglia cells, it has been reported that abnormal microglia has a significant association with neurological disorders (Saijo and Glass, 2011; Louboutin and Strayer, 2013; Nakagawa and Chiba, 2015), and dysfunction of the clock system is one of the risk factors for the psychiatric diseases; therefore, the microglia clock may provide valuable targets for the development of novel therapeutic agent for the neurological disorders, and further research on this topic will aid in understanding the function and dysfunction of the brain.

## Conclusion

**Figure 2.**
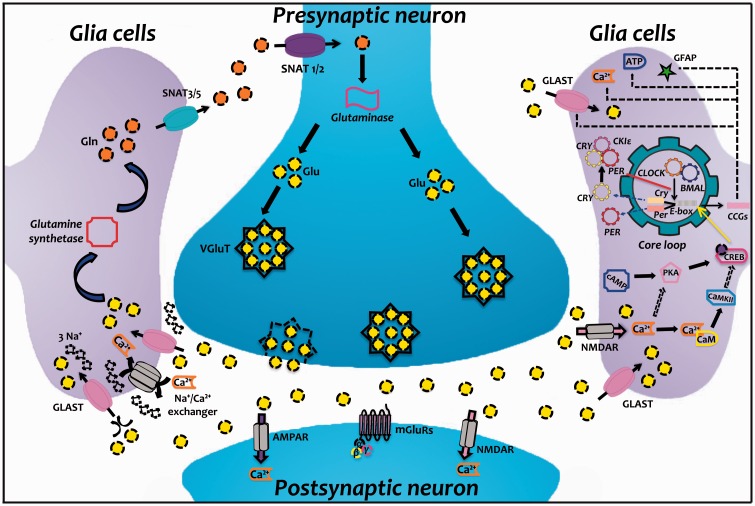
Model of a glutamatergic synapse and the molecular circadian clockwork. In the presynaptic neuron, glutamine (Gln) is converted to glutamate (Glu) by *Glutaminase* and packaged into synaptic vesicles by the vesicular glutamate transporter (VGluT). After its release into the extracellular space, Glu binds to ionotropic glutamate receptors (NMDAR and AMPAR) and metabotropic glutamate receptors (mGluRs) in the membranes of postsynaptic neuron and glia cells. Later, Glu is cleared from the synaptic space through excitatory amino acid transporters (EAATs) on neighboring glia cells (GLAST); this Glu uptake leads to Na^+^ influx, which activates the Na^+^/Ca^2+^ exchanger, increasing intracellular Ca^2+^ levels. Within the glia cell, Glu is converted to Gln by *Glutamine synthetase* and the Gln is subsequently released by system N sodium-coupled neutral amino acid transporters (SNAT3/5) and taken up by neurons through system A transporters (SNAT1/2) to complete the Glu-Gln cycle. Interestingly, Glu plays an important role in circadian rhythms since they express molecular oscillators. Glu activates NMDAR-induced Ca^2+^ influx, which together with other second messengers triggers the activation of diverse signal transduction cascades, including calmodulin kinase II (CaMKII) activity and cAMP-dependent protein kinase (PKA). Although the cross talk between these diverse cascades is not currently well known, it is plausible that a common mechanism involved in this pathway is the phosphorylation of the cAMP response element binding protein (CREB). In turn, pCREB activates *Per1* and *Per2* transcription (these genes are also activated by CLOCK/BMAL1 binding to E-box). Circadian transcription factors also regulate the expression of numerous proteins, molecules, and second messengers, including GLAST, GFAP, ATP, and Ca^2+^. Solid lines represent mechanisms that have been described experimentally, and dashed lines indicate possible additional links of this pathway. AMPAR, α-amino-3-hydroxy-5-methyl-4-isoaxazolepropionate receptor; ATP, adenosine triphosphate; BMAL1, brain and muscle ARNT-like protein 1; CaM, calmodulin; cAMP, cyclic adenosine monophosphate; CCGs, clock-controlled genes; CLOCK, circadian locomotor output cycles kaput; *Cry*, cryptochrome; GFAP, glial fibrillary acidic protein; GLAST, glutamate aspartate transporter; NMDAR, N-methyl-D-aspartate receptor; *Per*, period.

The presence of clock genes in glia cells has great importance for maintaining the homeostasis of the various functions performed by these cells (Figure 2). Due to the nature of glia cells, most of the changes reported by alterations in the expression of clock genes lead to problems related to imbalance of the glutamatergic system, leading to severe pathophysiological scenarios. Future work should focus on the role of glia in different aspects of circadian behavior and medical implications that could strengthen for our understanding the role of glia cells in brain physiology.
